# P02-02 Evaluating community-based programmes for health promotion: a novel approach considering the complexity perspective

**DOI:** 10.1093/eurpub/ckac095.021

**Published:** 2022-08-29

**Authors:** Irma Huiberts, Amika Singh, Frank van Lenthe, Mai Chinapaw, Dorine Collard

**Affiliations:** 1 Mulier Institute, Utrecht, The Netherlands; 2 Center for Physically Active Learning, Faculty of Education, Arts and Sports. Western Norway University of Applied Sciences, Sogndal, Norway; 3 Department of Public Health, Erasmus Medical Centre, Rotterdam, The Netherlands; 4 Faculty of Geosciences, Utrecht University, Utrecht, The Netherlands; 5 Department of Public and Occupational Health, Amsterdam Public Health Research Institute, Amsterdam UMC, Vrije Universiteit Amsterdam, Amsterdam, The Netherlands

**Keywords:** Community-based, children, youth, evaluation, complex

## Abstract

**Background:**

For the past decade, community-based programmes have been a popular strategy for physical activity promotion. The implementation of these programmes is a complex process, characterized by (a) objectives that vary locally, (b) adaptions to the programme over time in response to a community's shifting needs, challenges and opportunities, (c) emergent outcomes, and (d) non-linear causality. This poses several challenges for evaluation, as commonly used evaluation designs mainly focus on predetermined programme components and outcomes. Such a traditional evaluation approach may overlook necessary but unanticipated programme developments or outcomes and provide limited opportunity to learn from these. The aim of this study was to develop a novel evaluation approach that considers the complexity perspective, in order to evaluate a large community-based programme for children's physical activity and health promotion in the Netherlands.

**Methods:**

An exploratory review of theoretical and methodological literature regarding community-based health promotion, complexity theory, developmental evaluation and theory based evaluation was conducted. Based on this review a novel evaluation approach was developed.

**Results:**

The developed evaluation framework focusses on elements of the complex and adaptive implementation process of community-based health promotion. These include the local programme theory, implementation, adaption, the influence of context and feedback loops, and intended as well as emergent and unintended outcomes. By studying each of these elements in practice using innovative qualitative methods, including Ripple Effects Mapping and the Critical Event Card tool, principles that guide effective health promotion across community contexts can be extracted. Practice-based knowledge can subsequently be validated in other contexts.

**Conclusions:**

The proposed evaluation approach aims to inform both research and practice (local and national programme planners and policy makers), by considering the implementation of community-based programmes as a complex process in evaluation. Using this evaluation approach will provide insight in how community-based health promotion programmes impact communities, and which mechanisms underly success or failure. The proposed evaluation approach may be relevant for other physical activity promotion programmes or policies. Since considering complexity in evaluation is a relatively new challenge in public health, we believe it is essential to share and deliberate on innovative evaluation approaches and methods.

